# Broadband focusing and collimation of water waves by zero refractive index

**DOI:** 10.1038/srep06979

**Published:** 2014-11-10

**Authors:** Chi Zhang, C. T. Chan, Xinhua Hu

**Affiliations:** 1 Department of Materials Science, Laboratory of Advanced Materials and Key Laboratory of Micro and Nano Photonic Structures (Ministry of Education), Fudan University, Shanghai 200433, China; 2 Department of Physics and Institute for Advanced Study, Hong Kong University of Science and Technology, Clear Water Bay, Hong Kong, China

## Abstract

It is always a challenge to realize extreme and unusual values of refractive index for a broad range of frequencies. We show that when water is covered by a thick, rigid and unmovable plate, it behaves like a medium with zero refractive index for water waves at any frequency. Hence, by covering water with a plate of a concave or rectangular shape, water waves can be focused or collimated in a broad range of frequencies. Experiments were conducted to demonstrate these effects and results are in excellent agreement with numerical simulations.

Zero index of refraction is a useful concept for wave manipulation and has recently attracted much attention^1^,^2^,^3^,^4^,^5^,^6^,^7^,^8^,^9^,^10^,^11^,^12^,^13^,^14^,^15^,^16^,^17^,^18^,^19^,^20^. In a zero-index medium, waves possess infinite phase velocity, infinite wavelength, and do not experience any spatial phase change. Thus, the shape of the wavefronts leaving a zero-index medium depends only on that of the exit surface of the medium, which gives high flexibility in controlling the wavefronts of outgoing beams^1^,^2^,^3^,^4^. By filling waveguides with zero-index media, more exotic phenomena such as tunneling and superscattering can be observed^11^,^12^,^13^,^14^,^15^,^16^,^17^,^18^,^19^,^20^.

The zero refractive index has been initially pursued for electromagnetic waves^1^,^2^,^3^,^4^,^5^,^6^,^7^,^8^,^9^,^10^,^11^,^12^,^13^,^14^,^15^,^16^,^17^,^18^,^19^,^20^ and realized within the framework of effective medium theory^1^,^4^,^5^,^6^,^7^,^8^,^15^,^16^. By employing artificial periodic structures such as metamaterials^1^,^15^,^16^ and photonic crystals^4^,^5^,^6^,^7^,^8^, a zero refractive index can be achieved near some resonant frequencies. However, such zero-index media are constructed mainly for electromagnetic waves^1^,^2^,^3^,^4^,^5^,^6^,^7^,^8^,^9^,^10^,^11^,^12^,^13^,^14^,^15^,^16^,^17^,^18^,^19^,^20^, and partially for acoustic/elastic waves^21^,^22^,^23^,^24^,^25^. It remains unknown whether and how a zero index can be realized in water waves.

Unlike electromagnetic and acoustic/elastic waves, water waves are mechanical waves that propagate along the water surface and with the restoring force provided by gravity^26^,^27^. For a constant water depth *h*, the dispersion of linear water waves is given by 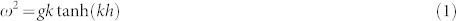
where *ω* is the angular frequency, *k* is the wavenumber and *g* is the gravitational acceleration^26^,^27^,^28^,^29^,^30^,^31^,^32^,^33^,^34^,^35^,^36^,^37^,^38^,^39^,^40^,^41^,^42^. In this paper, we theoretically and experimentally show that by covering water with a rigid and unmovable plate, a zero wavenumber and zero refractive index can be created for water waves at any frequency. As a result, interesting phenomena such as focusing and collimation of broadband water waves can be further realized by using different shapes of plates.

## Results

### System description, water wave equation and zero refractive index

**Figure 1:**
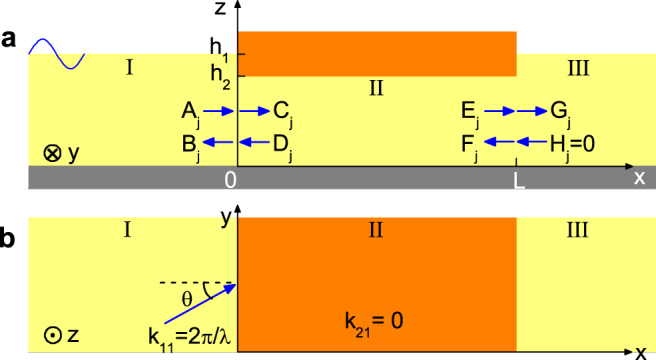
Schematic diagrams of a thick, rigid, and unmovable plate that is covered on the surface of water. (a) and (b) are the side and top views, respectively. The water has a depth *h*_1_ (*h*_2_) in the region without (with) the plate, and does not exist on the plate. The plate has a width *L* in the *x* direction and is infinitely long in the *y* direction. Water waves are incident onto the region with plate from the left and at angle *θ*.

We consider linear, inviscid and irrotational water waves in infinite extent of water as shown in Fig. 1. Set the *x*-*y* plane horizontal, the *z*-axis positive upward and the bottom of water in the *z* = 0 plane. The water depth is *h*_1_ in both region I with *x* < 0 and region III with *x* > *L*. In region II with 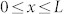, the water is covered by a thick, unmovable and rigid plate and has a depth of *h*_2_. For harmonic water waves, the velocity of water *v* is related to a potential Φ by *v*(*x*, *y*, *z*, *t*) = ∇Φ(*x*, *y*, *z*)*e*^−*iωt*^ 26,27. Φ satisfies the following equations 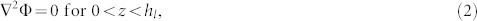

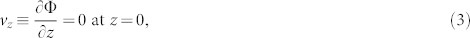
where the subscript *l* in *h_l_* is 1 and 2 in regions I/III and II, respectively. At the upper surfaces (*z* = *h_l_*) of regions I/III and II, the boundary conditions are 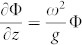 and 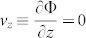, respectively, which can be written in a unified form, 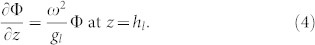
Here, *g_l_* = *g*_1_ = *g* in region I and *g_l_* = *g*_2_ → ∞ in region II.

Hence, the solution of Eqs. (2) – (4) is 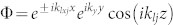, where 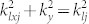, *ω*^2^ = *g_l_k_lj_* tanh(*k_lj_h_l_*) and 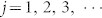. In regions I and III, the wavenumber *k*_1*j*_ is real (*k*_11_ > 0) for the fundamental mode (*j* = 1) and imaginary (*k*_1*j*_/*i* > 0) for higher-order modes (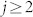). In region II, the fundamental mode has zero wavenumber (*k*_21_ = 0) and higher-order modes have imaginary wavenumber (*k*_2*j*_ = *i*(*j* − 1)*π*/*h*_2_ for 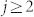). Hence, for phenomena dominated by the fundamental mode, region II can be regarded as a medium with zero refractive index (*n* ≡ *k*_21_/*k*_11_ = 0) for water waves.

### Coupled mode theory

The field 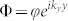 and *φ* in regions I, II, III can be written respectively as 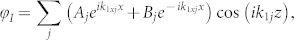

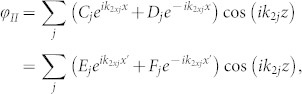

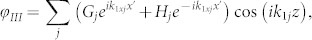
where *k_y_* = *k*_11_ sin *θ*, *θ* is the incident angle, *x*′ = *x* − *L* and (*A*, *C*, *E*, *G*) and (*B*, *D*, *F*, *H*) are the amplitudes of leftgoing and rightgoing waves, respectively. At the interface (*x* = 0) between regions I and II, the potential and velocity should be continuous [*φ_I_* = *φ_II_* and 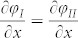 for 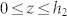 and 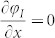 for 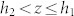]. Thus, we have 
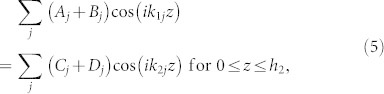

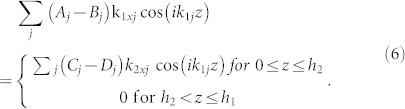


By multiplying Eq. (5) with 
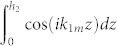
 and Eq. (6) with 
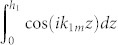
, we can have 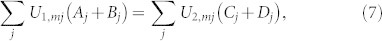

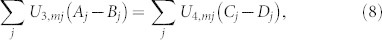
where 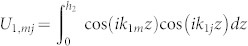, 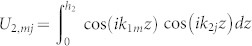, 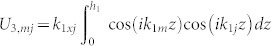 and 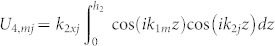 [see Methods].

### Transfer matrix formalism

Eqs. (7) and (8) can be rewritten as 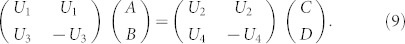
From Eq. (9), we have 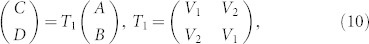
where 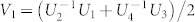 and 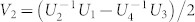.

In region II, we have 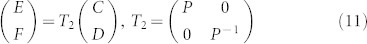
where 
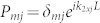
. At the interface between regions II and III, we can also obtain 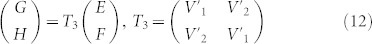
where 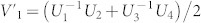 and 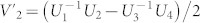 [see Supplementary information for derivations].

From Eqs. (10) – (12), we have 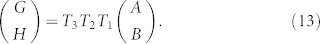
For incident waves from the left (*A_j_* = *δ*_1*j*_ and *H_j_* = 0), the transmission *t* = |*G*_1_/*A*_1_|^2^ and reflection *r* = |*B*_1_/*A*_1_|^2^ can then be calculated by Eq. (13). We note that Eqs. (10) – (13) represent the transfer matrix scheme for transmission calculations and can be rewritten as alternative forms of scattering matrix, which have higher stability in numerical calculations [see Supplementary information for details].

A finite number of plane waves (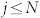) are adopted in numerical calculations. When *πL*/*h*_2_ > 1, the evanescent waves (with 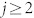) can be neglected (*i.e.* *N* = 1 is used) so that an analytic formula can be obtained for the transmission at normal incidence (*θ* = 0), 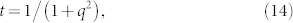
where 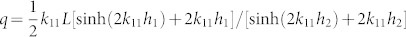 [see Supplementary information for derivations].

### Calculated transmission through water covered by a long rectangular plate

**Figure 2:**
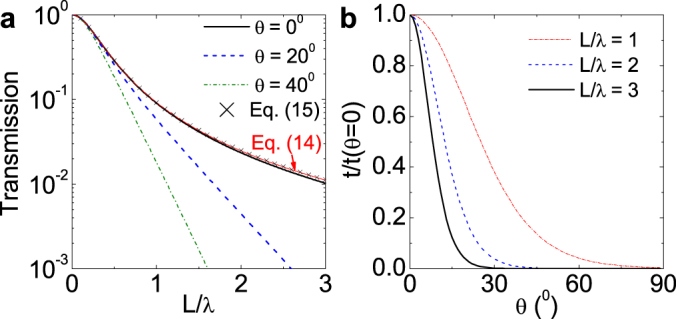
Transmission spectra for water waves through the water covered by a plate as shown in Fig. 1. The parameters are *h*_1_ = *h*_2_ = 6 mm and *L* = 5*h*_1_ = 30 mm. (a) Transmission as a function of reduced frequency *L*/*λ* at incident angle *θ* = 0^0^, 20^0^, and 40^0^. (b) Transmission as a function of angle *θ* at *L*/*λ* = 1, 2, and 3.

The above theory shows that water covered by a plate can be regarded as a zero-index medium (*k*_21_ = 0) for water waves and exhibit a relatively high transmission for long wavelengths (*L*/*λ* < 1). As an example, the transmission spectra are calculated for the case with *h*_1_ = *h*_2_ = 6 mm and *L* = 30 mm, as shown in Fig. 2a. To obtain convergent results, 5 plane waves (*N* = 5) have been adopted. It can be seen that for normal incidence (*θ* = 0), the accurate results can be well described by Eq. (14). A high transmission occurs for low frequencies (*L*/*λ* < 1). For oblique incidence, all the waves become evanescent (Im(*k*_2*xj*_) = *k*_11_ sin *θ* > 0) in region II, so that the transmission decays almost exponentially with increasing frequency [Fig. 2a]. As a result, at a given frequency, a relatively large transmission occurs only when the incident angle is around zero, and this phenomenon becomes more apparent with increasing the frequency [Fig. 2b].

Eqs. (2) – (4) are an accurate 3D theory for linearized water waves and can be approximately replaced by a 2D equation^31^,^37^, 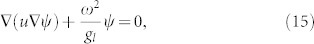
where *ψ*(*x*, *y*) is the potential Φ at the water surface (*z* = *h_l_*) and *u* = tanh(*kh*)/*k* is the effective depth. In Fig. 2a, we also show the results at normal incidence by Eq. (15). It can be seen that Eq. (15) can present almost the same results as those by Eq. (14).

### Experimental observations and numerical simulations of focusing effect

**Figure 3:**
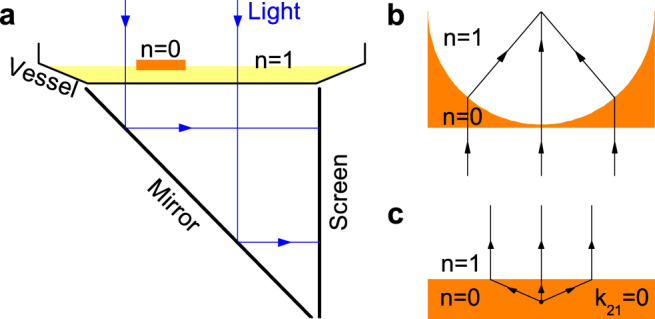
Schematic diagram of the experimental setup. (a) Water is placed in a vessel with a transparent bottom and partially covered by a rigid and unmovable plate. The vessel has slanted sides that do not reflect water waves. By using a mirror and collimated light, patterns of water waves can be projected onto a screen and recorded by a digital camera. (b) A concave plate and (c) a rectangular plate that are used to cover the water in (a).

The zero-index media can be applied to refract and focus waves^3^,^4^. For instance, one can focus plane waves with a concave lens made of a zero-index medium. Here, we conduct a water-wave experiment to demonstrate such a focusing effect. Our experimental setup is sketched in Fig. 3a. Water is placed in a vessel and partially covered by a concave, rigid and unmovable plate, which is obtained by cutting a half circle with radius of 20 cm on a 40 × 20.5 cm^2^ rectangular plate [Fig. 3b]. The water depths are 6 mm and 5 mm in the regions without and with the plate, respectively. The plate is impinged by a Gaussian water-wave beam with width of 28 cm. Since the vessel has a transparent bottom, patterns of water waves can be projected onto a screen and then recorded by a digital camera^32^,^35^. On the other hand, we also apply a finite-element method to simulate the experiment.

**Figure 4:**
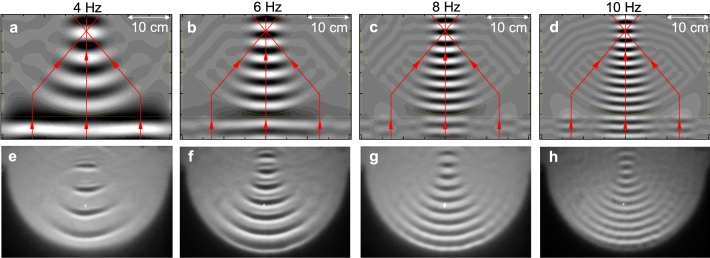
Wave patterns for impinging a Gaussian beam of water waves upon a water covered by a thick, rigid, unmovable, and concave plate. The plate is obtained by cutting a half circle with radius of 20 cm on a 40 × 20.5 cm^2^ rectangular plate. The water depths are 6 mm and 5 mm in the regions without and with the plate, respectively. (a) – (d) Simulated results at *f* = 4, 6, 8, 10 Hz. Black and white represent the upward and downward movement of water surface, respectively. The yellow lines outline the plates and the lines with arrows are analyses of refraction. (e) – (h) Experimental results at *f* = 4, 6, 8, 10 Hz.

Figures 4(a) –4(h) show the simulated and observed wave patterns for different frequencies. Good agreement is found between the theory and experiment. Here, water waves are normally incident on the flat side of the focusing lens (*i.e.* water covered by the concave plate), so that they do not refract at the entrance side. But when water waves leave the lens, they do refract at the exit side with a concave shape [Figs. 4a–4d]. All the outgoing waves are perpendicular to the exit side and thus directed to the focus of lens (*i.e.* the center of the half circle) [Figs. 4e–4h]. Since our zero index is independent on frequency, the focusing effect can be observed in a broad band of frequencies.

### Experimental observations and numerical simulations of collimation effect

**Figure 5:**
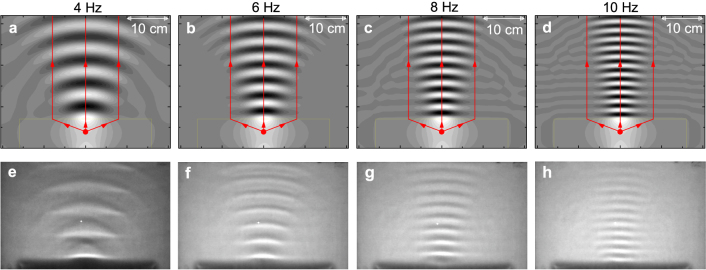
Wave patterns for placing a point source (monopole) of water waves in a water covered by a rigid, unmovable, and rectangular plate. (a) – (h) are the same as Figs. 4a–4h, except a point source and a 32 × 7 cm^2^ rectangular plate are applied. The red dots in (a) – (d) indicate the positions of point sources. The point source of water waves is experimentally generated by connecting an air tube with oscillating pressure to a hole through the plate.

In addition to focusing external water waves, the plate-covered water can also be applied to modify the emission property of an inner source, as shown in Fig. 5. Here, water is partially covered by a 32 × 7 cm^2^ rectangular plate that is drilled with a small hole. The hole is connected to an air tube with oscillating pressure, so that a point source of water waves can be generated in the plate. At the exit side of the plate, the emergence angle of water waves are always zero for arbitrary incident angle. As a result, a collimated beam can be observed in the water region without plate [Figs. 5a–5d]. Again, the experimental results are well described by the theory, showing that the water covered by the unmovable plate indeed possesses a zero index for water waves.

## Discussion

In the above experiments, the focusing and collimation effects were demonstrated by using a plates with size larger than the water depth. We note that the effects can also be realized by using a plate that has a size smaller than the water depth but larger than the wavelength. When the water depth is much larger than the wavelength (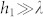), the velocity of water is almost zero near the bottom (*z* < *h*_1_ − 20*λ*). Hence, in numerical simulations, *h*_1_ and *h*_2_ can be replaced by *h*_1_ − *h_d_* and *h*_2_ − *h_d_*, respectively, where *h_d_* = *h*_1_ −min(*h*_1_, 20*λ*). By using five wave modes (*N* = 5), transmission spectra (similar to the curves in Fig. 2) can also be obtained.

Region II in our water-wave system, exhibiting an infinite effective gravitational acceleration (*g*_2_ = 0) and normal depth (*h*_2_ = *h*_1_), has its optical analogue that is a medium with zero magnetic permeability (*µ* = 0) and normal dielectric constant (*ε* = 1). Such a single zero (*µ* = 0) medium can be realized by using a photonic crystal with a square lattice of dielectric cylinders in air^43^. Near a particular frequency, the focusing and collimation effects have also been observed for electromagnetic waves. In contrast, since our infinite effective gravity is valid for water waves at any frequency, our effects can occur in a broad range of frequencies.

In summary, we have shown theoretically and experimentally that by covering water with a rigid and unmovable plate, a zero wavenumber and zero refractive index can be achieved for water waves in a broad range of frequency. By using plates with different shapes, interesting phenomena including focusing and collimation have been demonstrated in water waves. The results suggest a new mechanism for controlling the propagation of water waves and may find applications in wave energy focusing and extraction.

## Methods

### Calculations of *U*_1_, *U*_2_, *U*_3_ and *U*_4_

The matrices *U*_1_, *U*_2_, *U*_3_ and *U*_4_ can be calculated by using cos(*ik*_1_*z*) cos(*ik*_2_*z*) = [cos[*i*(*k*_1_ + *k*_2_)*z*] + cos[*i*(*k*_1_ − *k*_2_)*z*]]/2, 
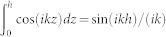
 for *k* ≠ 0, and 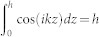 for *k* = 0.

### Simulations of focusing and collimation effects

Either Eqs. (2) – (4) or Eq. (15) can be simulated by using a finite-element method, so that Figs. 4a–4d and Figs. 5a–5d can be obtained.

## Author Contributions

X.H. and C.T.C. initiated the research. X.H. performed analytical derivations. C.Z. and X.H. performed numerical calculations and experiments. All the authors discussed the results and contributed to the writing of the paper.

## Supplementary Material

Supplementary Information Supplementary Information
