# Supplementary material for: Broadband focusing and collimation of water waves by zero refractive index
Source: Sci Rep. 2014 Nov 10;4:6979. doi: 10.1038/srep06979 (PMC4225562; doi:10.1038/srep06979)
Supplement: Supplementary Information [file srep06979-s1.pdf]

Broadband focusing and collimation of water waves by zero refractive index(Supplementary Information)

Chi Zhang,[1] C. T. Chan,[2] and Xinhua Hu[1,*]

[1]*Department of Materials Science, Laboratory of Advanced Materials, and Key Laboratory of Micro and Nano Photonic Structures (Ministry of Education), Fudan University, Shanghai 200433, China*

[2]*Department of Physics, Hong Kong University of Science and Technology, Clear Water Bay, Kowloon, Hong Kong, China*

I. Derivation of Eq. (12)

Eq. (9) can be rewritten as

$$\begin{pmatrix} B \\ A \end{pmatrix} = T_3 \begin{pmatrix} D \\ C \end{pmatrix}, \; T_3 = \begin{pmatrix} V_1' & V_2' \\ V_2' & V_1' \end{pmatrix}, \tag{S1}$$

where $V_1' = (U_1^{-1}U_2 + U_3^{-1}U_4)/2$ and $V_2' = (U_1^{-1}U_2 - U_3^{-1}U_4)/2$. By replacing (A, B, C, D) with (H, G, F, E) in Eq. (S1), we can obtain Eq. (12).

II. Scattering-matrix forms of Eqs. (10)-(13)

Eq. (9) can be rewritten as

$$\begin{pmatrix} C \\ B \end{pmatrix} = S^A \begin{pmatrix} A \\ D \end{pmatrix}, \; S^A = \begin{pmatrix} S_1^A & S_2^A \\ S_3^A & S_4^A \end{pmatrix} = \begin{pmatrix} U_2 & -U_1 \\ U_4 & U_3 \end{pmatrix}^{-1} \begin{pmatrix} U_1 & -U_2 \\ U_3 & U_4 \end{pmatrix}. \tag{S2}$$

Eq. (11) can be rewritten as

$$\begin{pmatrix} E \\ D \end{pmatrix} = S^B \begin{pmatrix} C \\ F \end{pmatrix}, \; S^B = \begin{pmatrix} S_1^B & S_2^B \\ S_3^B & S_4^B \end{pmatrix} = \begin{pmatrix} P & 0 \\ 0 & P \end{pmatrix}. \tag{S3}$$

Eq. (S2) can be rewritten as

$$\begin{pmatrix} B \\ C \end{pmatrix} = S^C \begin{pmatrix} D \\ A \end{pmatrix}, \; S^C = \begin{pmatrix} S_1^C & S_2^C \\ S_3^C & S_4^C \end{pmatrix} = \begin{pmatrix} S_4^A & S_3^A \\ S_2^A & S_1^A \end{pmatrix}. \tag{S4}$$

By replacing (A, B, C, D) with (H, G, F, E) in Eq. (S4), we can obtain

$$\begin{pmatrix} G \\ F \end{pmatrix} = S^C \begin{pmatrix} E \\ H \end{pmatrix}. \tag{S5}$$

Define the scattering matrices S^{BA} and S^{CBA} by

$$\begin{pmatrix} E \\ B \end{pmatrix} = S^{BA} \begin{pmatrix} A \\ F \end{pmatrix}, \; S^{BA} = \begin{pmatrix} S_1^{BA} & S_2^{BA} \\ S_3^{BA} & S_4^{BA} \end{pmatrix}, \tag{S6}$$

$$\begin{pmatrix} G \\ B \end{pmatrix} = S^{CBA} \begin{pmatrix} A \\ H \end{pmatrix}, \; S^{CBA} = \begin{pmatrix} S_1^{CBA} & S_2^{CBA} \\ S_3^{CBA} & S_4^{CBA} \end{pmatrix}. \tag{S7}$$

From Eqs. (S2), (S3) and (S6), we can have

$$\begin{aligned} S_1^{BA} &= S_1^B \left(1 - S_2^A S_3^B\right)^{-1} S_1^A, \; S_2^{BA} = S_2^B + S_1^B \left(1 - S_2^A S_3^B\right)^{-1} S_2^A S_4^B, \\ S_3^{BA} &= S_3^A + S_4^A \left(1 - S_3^B S_2^A\right)^{-1} S_3^B S_1^A, \; S_4^{BA} = S_4^A \left(1 - S_3^B S_2^A\right)^{-1} S_4^B. \end{aligned} \tag{S8}$$

By replacing (BA, B, A) with (CBA, C, BA) in Eq. (S8), we can have

$$\begin{aligned} S_1^{CBA} &= S_1^C \left(1 - S_2^{BA} S_3^C\right)^{-1} S_1^A, \; S_2^{CBA} = S_2^C + S_1^C \left(1 - S_2^{BA} S_3^C\right)^{-1} S_2^{BA} S_4^C, \\ S_3^{CBA} &= S_3^{BA} + S_4^{BA} \left(1 - S_3^C S_2^{BA}\right)^{-1} S_3^C S_1^{BA}, \; S_4^{CBA} = S_4^{BA} \left(1 - S_3^C S_2^{BA}\right)^{-1} S_4^C. \end{aligned} \tag{S9}$$

For incident waves from the left ($A_j = \delta_{1j}$ and $H_j = 0$), the transmission $t = |G_1|^2 = \left|S_{1,11}^{CBA}\right|^2$ and reflection $r = |B_1|^2 = \left|S_{3,11}^{CBA}\right|^2$ can then be calculated by Eq. (S7). We note that Eqs. (S2), (S3), (S5), and (S7) are the scattering-matrix forms of Eqs. (10)-(13). When $N > 1$, the scattering matrix scheme has higher stability in numerical calculations than the transfer matrix scheme with Eqs. (10)-(13).

III. Derivation of Eq. (14)

For $N = 1$, we have

$$\begin{aligned}
U_1 &= U_{1,11} = \int_0^{h_2} \cos(ik_{11}z)\cos(ik_{11}z)dz = \left[\sinh\left(2k_{11}h_2\right) + 2k_{11}h_2\right]/\left(4k_{11}\right),\\
U_2 &= U_{2,11} = \int_0^{h_2} \cos(ik_{11}z)\cos(ik_{21}z)dz,\\
U_3 &= U_{3,11} = k_{1x1}\int_0^{h_1} \cos(ik_{11}z)\cos(ik_{11}z)dz = k_{1x1}\left[\sinh\left(2k_{11}h_1\right) + 2k_{11}h_1\right]/\left(4k_{11}\right),\\
U_4 &= U_{4,11} = k_{2x1}\int_0^{h_2} \cos(ik_{11}z)\cos(ik_{21}z)dz = k_{2x1}U_2,\\
V_1 &= (U_2^{-1}U_1 + U_4^{-1}U_3)/2 = a\left(1+b\right)/2,\ V_1' = (U_1^{-1}U_2 + U_3^{-1}U_4)/2 = a^{-1}\left(1+b^{-1}\right)/2,\\
V_2 &= (U_2^{-1}U_1 - U_4^{-1}U_3)/2 = a\left(1-b\right)/2,\ V_2' = (U_1^{-1}U_2 - U_3^{-1}U_4)/2 = a^{-1}\left(1-b^{-1}\right)/2,\\
P &= P_{11} = e^{ik_{2x1}L},\ a = \frac{U_1}{U_2},\ b = \frac{U_2U_3}{U_1U_4} = \frac{k_{1x1}\left[\sinh\left(2k_{11}h_1\right) + 2k_{11}h_1\right]}{k_{2x1}\left[\sinh\left(2k_{11}h_2\right) + 2k_{11}h_2\right]}.
\end{aligned}$$

From Eq. (13), we have

$$\begin{aligned}
\begin{pmatrix} G \\ H \end{pmatrix} &= T_3T_2T_1\begin{pmatrix} A \\ B \end{pmatrix} = \begin{pmatrix} V_1' & V_2' \\ V_2' & V_1' \end{pmatrix}\begin{pmatrix} P & 0 \\ 0 & P^{-1} \end{pmatrix}\begin{pmatrix} V_1 & V_2 \\ V_2 & V_1 \end{pmatrix}\begin{pmatrix} A \\ B \end{pmatrix}\\
&= \begin{pmatrix} V_1'PV_1 + V_2'P^{-1}V_2 & V_1'PV_2 + V_2'P^{-1}V_1 \\ V_2'PV_1 + V_1'P^{-1}V_2 & V_2'PV_2 + V_1'P^{-1}V_1 \end{pmatrix}\begin{pmatrix} A \\ B \end{pmatrix} = \begin{pmatrix} T_{11} & T_{12} \\ T_{21} & T_{22} \end{pmatrix}\begin{pmatrix} A \\ B \end{pmatrix}.
\end{aligned}$$

Hence, the reflection coefficient can be obtained,

$$\begin{aligned}
r_A &= -\frac{T_{21}}{T_{22}} = -\frac{V_2'PV_1 + V_1'P^{-1}V_2}{V_2'PV_2 + V_1'P^{-1}V_1} = -\frac{\left(1-b^{-1}\right)P\left(1+b\right) + \left(1+b^{-1}\right)P^{-1}\left(1-b\right)}{\left(1-b^{-1}\right)P\left(1-b\right) + \left(1+b^{-1}\right)P^{-1}\left(1+b\right)}\\
&= -\frac{\left(1-b^2\right)\left(P-P^{-1}\right)}{\left(1-b\right)^2P - \left(1+b\right)^2P^{-1}} \overset{A1}{=} -\frac{\left(1-b^2\right)ik_{21}Lb^{-1}/2}{-1 + \left(1+b\right)^2 ik_{21}Lb^{-1}/2} \overset{A2}{=} -\frac{-ik_{21}Lb/2}{-1 + ik_{21}Lb/2} = \frac{iq}{-1+iq},\\
q &= \frac{1}{2}k_{21}Lb \overset{A2}{=} \frac{1}{2}\frac{k_{11}L[\sinh(2k_{11}h_1) + 2k_{11}h_1]}{\sinh(2k_{11}h_2) + 2k_{11}h_2},
\end{aligned}$$

where the condition of $A1$ is $P = 1 + ik_{21}L$ and $P = 1 - ik_{21}L$, and the condition of $A2$ is $b \to \infty$. The conditions of $A1$ and $A2$ can be satisfied at normal incidence ($\theta = 0$, $k_y = 0$, and thus $k_{21} = k_{2x1} \to 0$). Thus, we have

$$t = 1 - r = 1 - |r_A|^2 = \frac{1}{1+q^2}. \tag{S10}$$

We note that Eq. (S10) is Eq. (14).

---

* Electronic address: huxh@fudan.edu.cn
